# The role of edible bird’s nest and mechanism of averting lead acetate toxicity effect on rat uterus

**DOI:** 10.14202/vetworld.2019.1013-1021

**Published:** 2019-07-12

**Authors:** Abdulla A. Albishtue, Nurhusien Yimer, Md. Zuki A. Zakaria, Abd Wahid Haron, Abd Salam Babji, Adamu A. Abubakar, Falah H. Baiee, Hazem Kareem Almhanna, Bahaa H. Almhanawi

**Affiliations:** 1 Department of Veterinary Clinical Studies, Faculty of Veterinary Medicine, University Putra Malaysia, 43400 Serdang, Selangor, Malaysia; 2 Department of Anatomy and Histology, Faculty of Veterinary Medicine, University of Kufa, Najaf, Iraq; 3 Department of Veterinary Preclinical Sciences, Faculty of Veterinary Medicine, University Putra Malaysia, 43400 Serdang, Selangor, Malaysia; 4 Innovation Centre for Confectionary Technology (MANIS), School of Chemical Sciences and Food Technology, Faculty of Science and Technology, Universiti Kebangsaan Malaysia, 43600 Bangi, Selangor, Malaysia; 5 Department of Companion Animal Medicine and Surgery, Faculty of Veterinary Medicine, University Putra Malaysia, 43400 Serdang, Selangor, Malaysia; 6 Department of Veterinary Surgery and Radiology, Usmanu Danfodiyo University, Sokoto, Nigeria; 7 Department of Pathology, Faculty of Medicine and Health Sciences, University Putra Malaysia, 43400 Serdang, Selangor, Malaysia

**Keywords:** edible bird’s nest, growth factors, lead acetate, proliferating cell nuclear antigen, superoxide dismutase, uterus

## Abstract

**Aim:**

This study aimed to evaluate the protective effect of edible bird’s nest (EBN) supplement on the uteri of rats exposed to lead acetate (LA) toxicity.

**Materials and Methods:**

Five treatment groups were established as follows: Group 1 (C), which was given distilled water; Group 2 (T0), which was administered with LA (10 mg/kg body weight [BW]); and Groups 3 (T1), 4 (T2), and 5 (T3), which were given LA (10 mg/kg BW) plus graded concentrations of 30, 60, and 120 mg/kg BW of EBN, respectively. Rats were euthanized at week 5 to collect blood for superoxide dismutase (SOD) assay, and uterus for histomorphological study and expression analyses of epidermal growth factor (EGF), vascular endothelial growth factor (VEGF), and proliferating cell nuclear antigen (PCNA).

**Results:**

Results revealed that LA causes destruction of uterine lining cells and necrosis of uterine glands of exposed rats without EBN supplement while the degree of damage decreased among EBN treated groups; T3 showed the highest ameliorating effect against LA toxicity, as well as an increased number of uterine glands. Increased levels of SOD were also achieved in EBN supplemented groups than the controls. Results of immunohistochemistry showed significantly higher expressions of EGF, VEGF, and PCNA levels (p<0.05) in T3 compared to other treatments. EBN maintained upregulation of antioxidant – reactive oxygen species balance.

**Conclusion:**

The findings showed that EBN could ameliorate the detrimental effects of LA toxicity on the uterus possibly by enhancing enzymatic antioxidant (SOD) activity as well as expressions of EGF, VEGF, and PCNA with cell proliferation roles.

## Introduction

Industrialization and urbanization increase the risk of daily exposure to a variety of chemical contaminants with adverse health effects. Lead is one of the heavy metal environmental pollutants known. Lead acetate (LA) can accumulate in the tissue of human and animals with a long half-life time [1-3]. Lead is well known for causing a range of physiological, behavioral, and biochemical dysfunction in animals and humans including the reproductive system, hematopoietic system, central and peripheral nervous system, kidneys, liver, cardiovascular system, and endocrine glands [4]. In the gastrointestinal tract, lead is first absorbed by red blood cells and circulated to all vascular organs [5]. Children are particularly susceptible to lead exposure as a result of high gastrointestinal absorption, and the permeable blood-brain barrier [1]. The LA is considered as one of the critical causes responsible for disturbances of oxidative stress, antioxidant in balance that leads to increases in reactive oxygen species (ROS), nitric oxide species (NOS), and lipid peroxidation markers such as malondialdehyde and inhibits antioxidant enzymes such as catalase, superoxide dismutase (SOD), and glutathione. It is, therefore, essential to conduct investigations on LA-induced mechanisms of fertility impairment at the genetic and biochemical level [6].

Lead is considered among the significant contributors of infertility and consequential productivity losses in animals, malnutrition, and ovulatory or hormonal imbalances [7,8]. However, information on the impact of environmental toxicants on female reproduction and how these affect breeding is almost obscure. Nevertheless, in experimental animals, long-term exposure to lead may result in inhibition of ovulation, follicular growth, and menstruation that is regulated by the secretions of uterine glands as reported in monkeys [9,10]. Moreover, it can cause a delay in the vaginal opening of pubertal rats [11] and a decrease in the frequency of implanted ova and pregnancies in mice [12]. Recently, the use of medicinal food with antioxidant activity has been introduced to the physiotherapists to protect them from heavy metal toxicity [13]. There has been increasing interest among therapy researchers to use edible bird’s nest (EBN) that has to reach nutritional content (water-soluble protein, carbohydrate, iron, inorganic salt, and fiber) and medicinal function (anti-aging, anticancer, immunity enhancing, and antioxidant) for protection against heavy metal toxicity. EBN has been reported to have many therapeutic effects. One of its most important traditionally believed benefits that need scientific approval is its enhancing effect on sexual ability or reproduction.

The objective of this study was to assess the protective effect of EBN pre-treatment against LA toxicity effect on female reproduction using a rat model. We fed EBN extracts to rats at different concentrations, conducted histopathological and histomorphometric analyses of uterine tissues, and detected the expression of genes of epidermal growth factor (EGF), vascular endothelial growth factor (VEGF), and proliferating cell nuclear antigen (PCNA).

## Materials and Methods

### Ethical approval

All procedures were performed according to Institutional Animal Care and Use Committee guideline, Universiti Putra Malaysia (UPM) (Project approval number: UPM/IACUC/AUPR009/2016).

### EBN preparation

EBN was purchased from Nest Excel Resources Sdn Bhd, maintained at 25°C-30°C. EBN extract was prepared in accordance with Chinese tradition as indicated by the local suppliers. The samples were cleaned, dried at room temperature, and ground into powder using a mixer (BUCHI-400, Switzerland). The ground EBN extract was maintained at 4°C. EBN solution was prepared by dissolving 1 g of EBN powder in 100 mL of distilled water, followed by heating in a water bath at 60°C for 45 min. Finally, the EBN solution was cooled down to room temperature and administered to the rats at doses based on their body weights (BWs).

### Preparation of LA solution

LA with a molecular formula of Pb (C_2_H_3_O_2_)_2_ was purchased commercially from Oxford Lab. Co., India (CAS: 6080-56-4). A 1% (w/v) solution of LA in distilled water was initially prepared, and individual rats of the treated groups were given orally at a dose of 10 mg/kg of BW using a gavage tube (China, Straight, 18 Gauge).

### Animals and experimental design

This study embodied 30 female Sprague–Dawley rats (aged 12 weeks) from the Animal Resource Center. The rats were housed in cages in groups for an acclimation period of 7 days with free access to water and standard rat diet (Gold Coin Brand Animal Feed). After 7 days of acclimation, rats were randomly grouped into five groups (six animals/groups) and subjected to LA (10 mg/kg BW) according to Assi *et al*. [8] and *in vivo* supplement of EBN according to Table-1 for a period of 5 weeks. The choice of the doses of EBN was according to a previous report by Albishtue *et al*. [14]. During the administration period, the rat’s BW in each group was measured and recorded every week. At the proestrus stage, the rats were euthanized at stipulated dates (5 weeks) by CO_2_ asphyxiation method following a general anesthesia procedure, which included injection of 30 mg ketamine/kg BW and 10 mg xylazine/kg BW for blood collection according to Albishtue *et al*. [14].

**Table 1:** Animal grouping and toxicity of LA and treatment regime of EBN administered by gavage needle.

Group	Group assigned	Type of feed(dose)
Control	C	Normal diet(ND) + Normal saline(1 mL)
Treated	T0	ND + LA(10mg/Kg BW)
	T1	ND + EBN(30mg/Kg BW)+ LA (10 mg/Kg BW)
	T2	ND + EBN(60mg/Kg BW)+ LA (10 mg/Kg BW.)
	T3	ND + EBN(120mg/Kg BW)+ LA (10 mg/Kg BW)

ND=Normal diet, LA=Lead acetate, EBN=Edible bird’s nest, BW=Body weight

### Determination of estrous cycle phases and synchronization

At the start of the experiment, the rats were synchronized using two intraperitoneal doses of 0.5 mg of Estrumate per rat 3 days apart [15]. Vaginal cytology was used to monitor the estrous cycle, according to Albishtue *et al*. [16]. The percentages of different types of cells in the vaginal smear, according to McLean *et al*. [17], determine stage of the cycle.

### Macroscopic and microscopic examinations of the reproductive tract

After sacrificing the anesthetized rats, their uteri were excised, weighed, and measured in length. Any evident abnormality found during the gross examination was recorded. The histology samples were fixed in 10% formalin for 24 h, sectioned, and stained using hematoxylin and eosin. The samples were observed under a microscope for histological changes, including those in the endothelial lining and the number of uterine glands [16,18]. Sections were cut from the central region to the peripheral region. The thicknesses of the luminal epithelium (LE), glandular epithelium (GE), and endothelium, and the number of uterine glands were determined using the Medical Image Analysis software (Motic Image plus 2.0, McAudi Industrial Group Co., Ltd, China).

### Analyses of the expressions of EGF, PCNA, and VEGF in uterine tissues by immunohistochemistry

The protein expression was investigated by immunohistochemistry, according to Albishtue *et al*. [19].

### Determination of SOD concentrations

Plasma samples were also obtained to analyze the enzymatic antioxidant, SOD activity according to the method as described by Schmidt *et al*. [20] and Albishtue *et al*. [16]. The SOD activity was determined in the plasma using the Enzychrom^™^ SOD Assay Kit (Bioassay System, San Francisco Bay Area, USA), which assesses the percentage of superoxide radicals that undergo dismutation in a given sample [20].

### Statistical analysis

All results were expressed as means (M) ± standard error of the mean (SE) and analyzed with GraphPad Prism 6.0 (GraphPad Software, San Diego, California). The data obtained were checked for normal distribution using the Shapiro–Wilk test. One-way analysis of variance (ANOVA) with Tukey multiple comparison *post hoc* test was used to compare the uterine BW ratios (UBWR) and lengths, numbers of uterine glands and SOD. Meanwhile, two-way ANOVA with Bonferroni’s multiple comparison tests was employed to compare the BWs. Comparison between thicknesses of the LE, GE, and endothelium; expression levels of growth factors, and PCNA, and steroid receptors were made using a Kruskal–Wallis (non-parametric) test. p<0.05 was considered statistically significant.

## Results

### Effect of EBN on estrus cycle, BW, and the UBWR of rats that were exposed to LA toxicity

**Figure-1:**
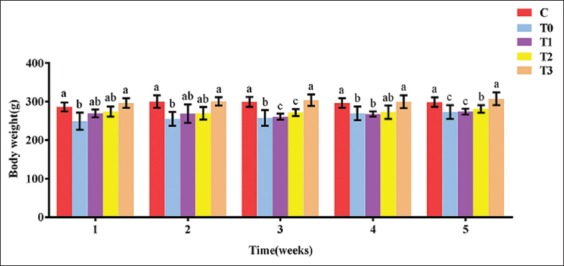
Effect of edible bird’s nest on body weight of rats exposed to lead acetate toxicity.

**Figure-2:**
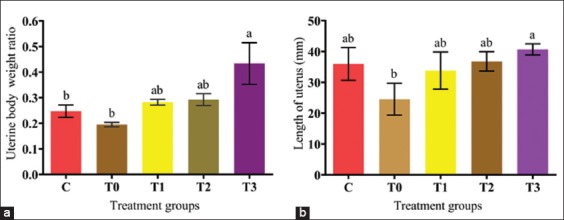
(a and b) Effect of edible bird’s nest on uterine-body weight ratio and uterine length of rats exposed to lead acetate toxicity.

Although rats exposed to LA had normal (4-5 days) estrus cycle and had no changes in the cell shapes of vaginal smear, there were significant changes in the BWs of rats in all groups throughout the experimental period. At week 5, the BW was lower in the positive control (T0) and higher (p<0.05) in the T3 group compared to Group C (Figure-1). Furthermore, the UBWRs and lengths were lower in the positive control (T0) and higher (p<0.05) in the T3 group compared to treatment groups and control. The changes significantly increased in the treatment groups and attained the highest value (p<0.05) in T3 (Figure-2a and b). The uterine weights and lengths increased dose-dependently with EBN.

### Histopathological findings in the uteri of rats that were exposed to LA and supplemented with EBN

**Figure-3:**
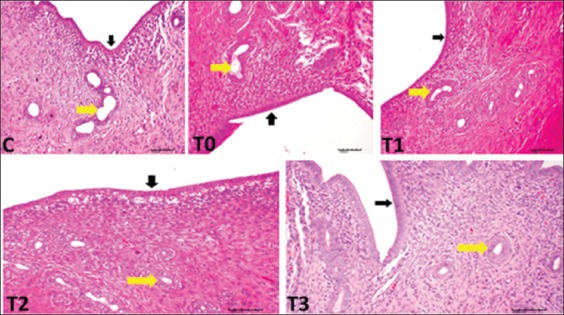
Histologic sections from adult rat uteri exposed to lead acetate toxicity. Black arrows and yellow arrows indicate uterine epithelial lining and uterine glands, respectively. Noticed necrosis of uterine glands and destruction of uterine lining cells in positive control T0, T1 and T2.

**Figure-4:**
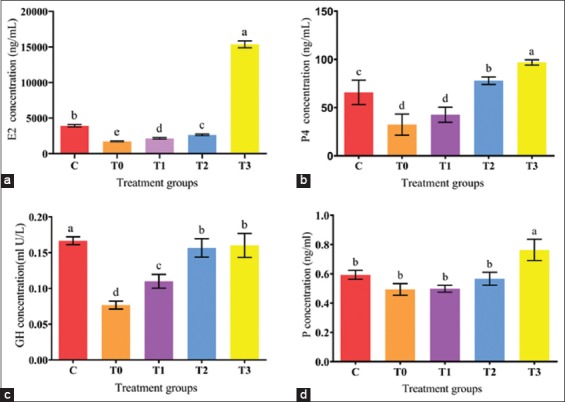
(a-d) Histomorphometric parameters evaluated in the rat uterus, measured during the proestrus phase. Note the lesser thickness of the endometrium and decrease numbers of uterine glands in T0. While, T3 showed greater thickness of the endometrium and increased numbers of uterine glands compared to other treatment groups.

There were no apparent gross pathological lesions found in the uterus. Nevertheless, histopathological inspection of uteri of different groups (except C and T3 groups) showed lead-induced inflammatory alterations, which were characterized by narrowing of the uterine lumen, atrophy of the endometrium, vacuolar degeneration in endometrial epithelial cells, damaged and decreased number of endometrial glands, and increased filtration of inflammatory cells. However, T3 showed the normal uterine structure as the control (Figure-3). The microscopic examination of the uteri also showed remarkable development and a greater quantity of endometrial structures in T3 samples compared with the T0 and the other groups (Figure-4).

### Effect of EBN on expressions of EGF, PCNA, and VEGF in the uteri of rats that were exposed to LA

**Figure-5:**
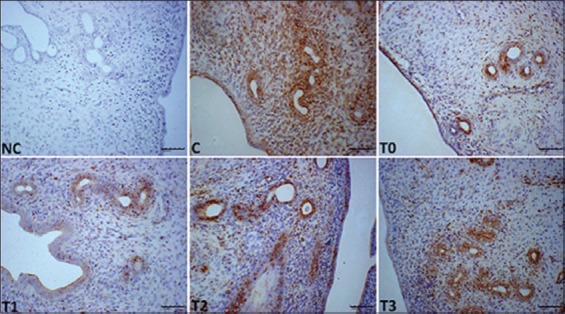
Photomicrograph sections of the uteri of rats of different experimental groups (C, T0, T1, T2, and T3) exposed to LA and treated with different doses of edible bird’s nest showing expression of epidermal growth factor (EGF) (200×). Notice low staining in T0 and T1 compared to other treatment groups. Photomicrographs labeled with NC represents control stains without antibody and immunity reaction for EGF groups. It shows higher EGF expression observed at T3 compared to other treatment groups.

**Figure-6:**
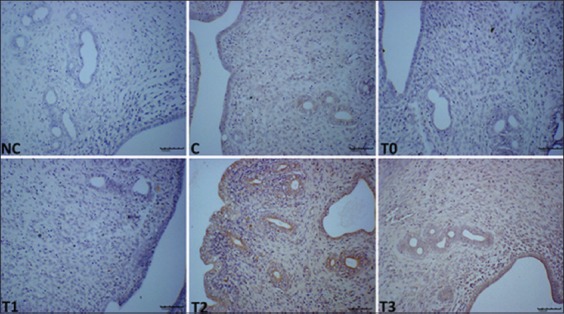
Photomicrograph sections of the uteri of rats of the different experimental groups (C, T0, T1, T2, and T3) exposed to lead acetate toxicity and treated with different doses of edible bird’s nest showing expression of vascular endothelial growth factor (VEGF) (200×). T0 and T1 have not staining for VEGF and higher VEGF expressions were observed in stromal cells, uterine luminal epithelium, and uterine glandular epithelium in T2 and T4 compared to control. Photomicrographs labeled with NC represents control stains without antibody and immunity reaction for VEGF groups. It shows higher VEGF expression observed at T3 compared to other treatment groups.

**Figure-7:**
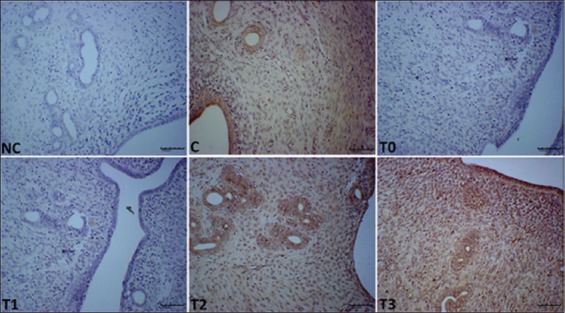
Photomicrograph sections of the uteri of rats of the different experimental groups (C, T0, T1, T2, and T3) exposed to lead acetate toxicity and treated with different doses of edible bird’s nest showing expression of proliferating cell nuclear antigen (PCNA) (200×). T0 and T1 have not staining for PCNA and higher expressions were observed in stromal cells, uterine luminal epithelium, and uterine glandular epithelium in T2 and T3 compared to control. Photomicrographs labeled with NC represents control stains without antibody and immunity reaction for PCNA groups. It shows higher PCNA expression observed at T3 compared to other treatment groups.

Representative immunohistochemistry photomicrographs that show the expression of EGF, the histological sections of the uteri of all experimental groups as well as their differences in scores are depicted in Figure-5 and Table-2. The expression level of EGF in the stromal cells, LE, and GE were lower (p<0.05) in T0 and T1 but higher (p<0.05) in T3. T2 is comparable with T3. VEGF and PCNA levels were different in groups, and a significant statistical difference was observed among the groups. The T3 and T2 groups had the highest VEGF and PCNA expression levels (p<0.05) than the other treatment groups and control, and no staining for VEGF was observed in T0 and T1 (Figures-6 and 7, and Table-2) groups.

**Table 2:** Expressions of EGF, VEGF, and PCNA in the LE, GE, and stromal cells of uteri of rats treated with LA and different doses of EBN, and sacrificed at proestrus stage of the estrous cycle.

Parameters	C	T0	T1	T2	T3
EGF in LE	1.5±0.08^a^	0.5±0.22^d^	0. 5±0.1^c^	0.8±0.1^bc^	1.0±0.0^b^
EGF in GE	2.0±0.14^a^	0.8±0.14^c^	1.0±0.07^c^	1.3±0.04^bc^	1.5±0.0^b^
EGF in S	2.0±0.14^a^	0.5±0.14^c^	0.5±0.14^c^	0.75±0.0^bc^	1.5±0.0^b^
VEGF in LE	1.0±0.07^c^	0	0	2.0±0.14^b^	2.5±0.07^a^
VEGF in GE	1.5±0.14^c^	0	0	2.0±0.14^b^	3.0±0.0^a^
VEGF in S	1.0±0.07^c^	0	0	1.5±0.014^b^	2.5±0.07^a^
PCNA in LE	1.5±0.14^c^	0	0	2.0±0.14^b^	3.0±0.00^a^
PCNA in GE	1.5±0.14^c^	0	0	2.0±0.14^b^	3.0±0.00^a^
PCNA in S	1.5±0.14^c^	0	0	2.5±0.07^b^	3.0±0.00^a^

LE=Uterine luminal epithelium, GE=Uterine glandular epithelium, S=Stromal cells. Data are expressed as means±standard error (SE). Different letters^a,b^ and ^c^denotes significant difference at p<0.05. Note NC=Control without antibody. G1=Control; G2=EBN (30 mg/Kg BW); G3=EBN(60 mg/Kg BW); G4=EBN(120 mg/Kg BW). EBW=Edible bird’s nest, PCA=Proliferating cell nuclear antigen, VECF=Vascular endothelial growth factor, EGF=Epidermal growth factor

### Antioxidant enzyme SOD concentrations

**Figure-8:**
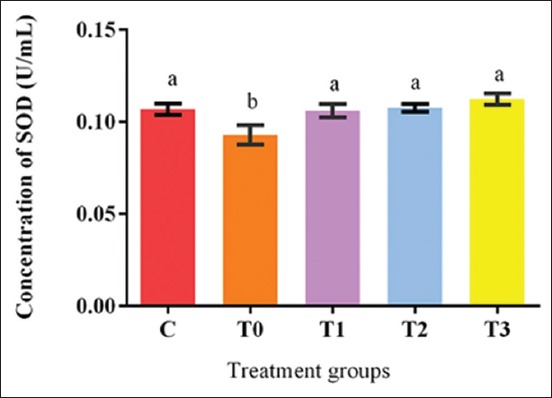
Effect of edible bird’s nest on antioxidant enzyme (superoxide dismutase) activities in plasma of lead acetate exposed and EBN treated rats.

The plasma SOD activities obtained from all the experimental groups of the present study are summarized in Figure-8. The increase in SOD activity in the treatment groups was dose-dependent at week 5. The concentrations of SOD were lower (p<0.05) in the EBN unsupplemented group T0 and higher in T3. However, the difference between the groups other than T0 was insignificant (p>0.05).

## Discussion

Following exposure, lead is deposited predominantly in the human bones [21,22]. Previous studies suggested that re-released lead to general circulation can be induced by various factors such as age or hormone production [23,24]. Therefore, lead characterized by having long half-lives [1-3]. Previous studies have generally reported deleterious effects of LA on the mature female reproductive organs and fertility of rats [7,25]. Vaginal histological data of the present study showed that the estrus cycle remains normal despite exposure to LA. In addition, the previous study showed that cycles were normal for the treated monkeys before exposure to lead and that three of four-cycle characteristics remained normal during the first lead exposures. However, chronic exposure to lead may cause alteration of menstruation. The lead-treated monkeys had longer and more variable intercycle intervals, as well as fewer days of vaginal bleeding than control [10]. The administration of different doses of LA was shown to cause reduced BW, uterine weight, and a decrease in the frequency of ova and pregnancies in mice [12]. Although a recent study highlighted the impact of EBN supplement on normal rats’ uteri [16], there are no reports on the effect of EBN on LA-induced uterine injuries.

In this study, LA caused a reduction in BW and the UBWR. Moreover, histological examination of the uteri of LA exposed rats without EBN supplement showed necrosis and reduced number of uterine glands and destruction of uterine lining cells with increased infiltration of inflammatory cells. This observation is in agreement with Eugenia *et al*. [25]. However, EBN supplemented groups (especially T3group) showed normal uterine structures and morphological parameters of uteri were higher than T0 and the control, despite exposure to LA toxicity. This interestingly may imply that EBN at a dosage of 120 mg/Kg BW supplement played not only protection against the adverse effect of the LA but also favored the proliferation of uterine structures more than the negative control group (with no EBN and LA treatment).

Similarly, previous comparative studies revealed that EBN had increased proliferation rate of the human colonic adenocarcinoma cell line (Caco-2 cells) about 135%-215% compared to the control and reduced the percentage of tumor necrosis factor-alpha *in vitro* [26,27] (Aswir and Wan Nazaimoon, 2011; Vimala *et al.*, 2012). The authors suggested that EBN extract may have anti-inflammatory properties as a result of inhibition of nitric oxide production [27]. According to Abidin *et al*. [28], a low concentration of EBN (0.05% or 0.1%) has the capacity to improve the cell proliferation of corneal keratocytes, derived from New Zealand white rabbits, in both serum-containing and serum-free media. The increased proliferation was explained by the presence of EGF in the EBN extract [29]. These properties of EBN were considered as a breakthrough for promoting the healing process in corneal wounds. Along with the conclusion of Wong *et al*. [30], several compounds of Chinese herbal medicines, including EBN, have been found to boost the proliferation of adult’s stem cell during tissue regeneration.

EBN contains VEGF and Interleukin-6 (IL-6) that prevents apoptosis of embryonic neurons by interfering with the activation of caspase three, leading to suppress the apoptotic cells [31]. An earlier study on the composition of EBN has reported the presence of reproductive hormones including testosterone, E2, P4, luteinizing hormone (LH), Follicle-stimulating hormone (FSH) as well as prolactin [32]. Previous studies have also suggested that there is a synergistic relation among VEGF, FSH, and estradiol in preventing apoptosis, inhibiting caspase 3 activation, and stimulating proliferation [33-35]. Thus, the effect of EBN observed on the uterus in the current study might be attributed to some of these critical bioactive compounds that it contains. The nutritional composition of EBN in order of amount includes protein, carbohydrate, ash, and lipid [32]. EBN has an esteemed nutritional value due to its water-soluble contents such as proteins, carbohydrates, iron, inorganic salts, and fiber [36]. Keeping with the composition of EBN, sialic acid also has some molecular mechanisms that cause cell proliferation. Sialic acid binding protein expression in the endometrium of human, which is regulated by the hormone estradiol has been reported earlier [37]. The presence of EGF-like component found in EBN has also been associated with its role in cell division, growth, and enhancement of tissue regeneration. This phenomenon has been considered as one of the reasons for EBN’s rejuvenating properties [38].

Histologically, the uterine cells and glands of rats exposed to LA showed increased activity when treated with EBN. Lead may produce a deleterious effect on *in utero* physiological functions by damaging uterine glands that lead to altered endometrial glandular secretions such as enzymes, growth factors, cytokines, as well as hormones and transport proteins essential for the development of conceptus [39]. Furthermore, PCNA density increased in the uterus, which is a sign of DNA synthesis and cell proliferation. These results provide additional evidence to the potential role of EBN in reproduction and fertility. The EGF, VEGF, and IL-6 play essential roles in cellular processes and are intercellular mediators that control growth, survival, and cellular differentiation, and function [40,41]. This notion agrees with our present results, where the significant expression of EGF was associated with the abundant proliferation of LE, GE, and uterine glands and the thickening of the endothelium. Similar to the study of Roh *et al*. [42], the current study confirmed that EBN enhances EGF, VEGF, and PCNA expression in the GE, USE, and uterine stromal cells, thereby indicating the proliferative effect of EBN. Moreover, VEGF is an angiogenic factor in the endometrium and hence is vital to the development, maintenance, and degradation of the structure. The VEGF is also a major factor for intensifying the vascularization of uterine glands and stromal cells and thus improves nutrient supply. The link between increased VEGF expression and EBN supplementation reveals how EBN promotes the proliferation of stromal cells, LE, and GE. The VEGF is related to the actions of LH and angiopoietin produced in luteinizing cells [43-45]. In the present experiment, EBN also increased expressions of EGF, PCNA, VEGF in stromal cells, and glandular and uterus epithelium. The increased expressions of EGF, VEGF, and PCNA suggest that EBN has a proliferative effect and therapeutic effect against heavy material toxicity [46].

Many disease conditions are related to disturbances of antioxidant enzymes such as catalase, SOD, glutathione as well as increases in ROS, NOS, and lipid peroxidation markers such as malondialdehyde. Supplementation with EBN as an antioxidant has been observed to aid in disease prophylaxis as well as treatment [16,47]. In the current study, EBN prevented alteration of the cellular redox state induced by LA by increasing antioxidant capacity and enhancing one of the enzymes involved in antioxidant defense that is SOD, which functions as blockers of the free radical process. The induction of antioxidant enzymes may result in enhancing membrane integrity, thereby increasing the resistance of the membrane to metal exposure. As observed from the findings from the present study, EBN supplement as low as 30mg/kg BW is sufficient enough to prevent disturbance of the redox system due to LA toxicity by enhancing the enzymatic antioxidant defense.

## Conclusion

The present study revealed that oral supplementation of EBN at the dose of 60-120 mg/kg BW is capable of protecting and preventing alterations in the reproductive system histomorphology and function due to LA toxicity through an integrated mechanism of maintaining antioxidant – ROS balance and upregulation of genes of EGF, VEGF, and PCNA.

## Authors’ Contributions

NY designed and supervised the study. AAA conducted the research work. AAA, FHB, AAAb, and BHA performed the investigations. ASB prepared Edible bird’s nest. MZAZ and AWH conceptualized and contributed to the study design. HKA discussed and reviewed the manuscript. NY and AAA analyzed the data and drafted the manuscript. All authors read and approved the final manuscript.
